# Correlation of Renal Scarring to Urinary Tract Infections and Vesicoureteral Reflux in Children

**DOI:** 10.1155/2022/9697931

**Published:** 2022-04-26

**Authors:** Hamdy Aboutaleb, Tamer A. Abouelgreed, Hala El-Hagrasi, Diaa Bakry Eldib, Mohamed A. Abdelaal, Mohamed Amin El Gohary

**Affiliations:** ^1^Department of Urology, Menoufia University, Shibin Al Kawm, Egypt; ^2^Burjeel Hospital, Abu Dhabi, UAE; ^3^Department of Urology, Al-Azhar University, Cairo, Egypt; ^4^Department of Urology, Gulf Medical University, Ajman, UAE; ^5^Department of Pediatric, Suez Canal University, Sheikh Zayed City, Egypt; ^6^Radiodiagnosis Department, Faculty of Medicine, Benha University, Benha, Egypt

## Abstract

**Objective:**

To study the association of the grade of vesicoureteral reflux (VUR) and urinary tract infections (UTI) with renal scarring at the first clinical presentation of patients who underwent antireflux surgery. *Materials and methods*. Between 2015 and 2020, 150 patients (194 units) who underwent antireflux surgery had dimercaptosuccinic acid (DMSA) renal scans preoperatively. Patients were classified into the nonscar and scar groups according to DMSA scan results. Moreover, cases were classified into afebrile UTI, febrile UTI, and antenatal hydronephrosis (ANH) according to the mode of presentation. We correlated the mode of presentation and the grade of VUR to the presence/absence of renal scars in both groups.

**Results:**

The mean follow-up was 45 months preoperatively. The mode of presentation was afebrile, febrile UTIs, and antenatal hydronephrosis in (50, 14), (20, 46), and (10, 10) patients in the nonscar and scar groups, respectively. Of the 20 patients who presented ANH, 10 (50%) had scars. Clinical presentation was correlated to the presence of renal scarring and its degree. The scar group had significantly higher grades of VUR than the nonscar group (grades I–II (50 units versus 10 units), grade III (28 units versus 40 units), and grade IV–V (22 units versus 44 units) for the nonscar versus scar groups, respectively (*p*value <0.001).

**Conclusion:**

Renal scarring is associated with higher grades of reflux and urinary tract infections. We advocate further research investigating infants who had UTIs with or without fever for early detection of reflux.

## 1. Introduction

Vesicoureteral reflux (VUR) is a common problem in children and is mainly presented with febrile urinary tract infections (FUTIs) [1]. FUTI is one of the most common serious febrile illnesses in children, and its incidence in the first 6 years of life accounts for up to 8.4% of girls and 1.7% of boys. After the assessment of FUTI, approximately 30 to 50% of children who underwent voiding cystourethrograms have VUR [2]. The association between VUR, UTIs, and renal damage is well-known [3]. Reflux nephropathy which is mainly associated with high-grade reflux causes renal scars and can end in end-stage renal disease in up to 3% to 25% of cases [4]. Reflux nephropathy develops as a result of high-grade fetal reflux in the absence of UTIs and could cause renal scars as well [4]. It is difficult to determine whether these scars are due to fetal nephropathy or FUTIs. We hypothesize that there is an association between the higher grades of reflux and FUTIs to renal scarring at the first clinical presentation of patients who underwent antireflux surgery.

## 2. Patients and Methods

**Figure 1:**
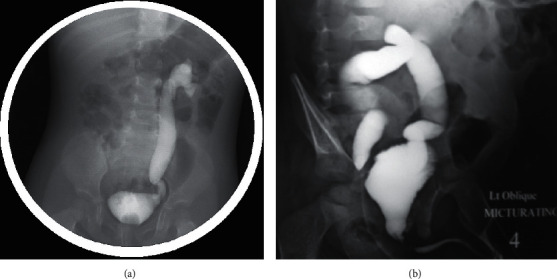
(a) Voiding cystourethrography image shows grade V left vesicoureteral reflux. (b) Voiding cystourethrography image shows bilateral vesicoureteral reflux grade V in a newborn.

After obtaining institutional review board approval, we conducted a retrospective study and reviewed patients' charts who underwent antireflux surgeries from January 2015 to May 2020. We recruited 150 patients (194 units) who were diagnosed with VUR. We only included children with primary VUR who presented to our institution and were evaluated using voiding cystourethrography (VCUG) and dimercaptosuccinic acid (DMSA) renal scan. We excluded patients who had multiple nonurological congenital anomalies and patients with secondary VUR such as those with myelomeningocele, ureterocele, urethral valves, and duplex kidney. We recorded patients' characteristics including age, gender, mode of presentation, side of VUR as well as laterality. Modes of presentation were FUTI, afebrile UTI, and antenatal hydronephrosis (AHN). Urinary tract infection was defined as the growth of at least 105 colony-forming units per milliliter of a single bacterial species from midstream or catheter specimens. When UTIs are associated with fevers, more than 38 degrees is defined as febrile UTIs. All included patients were evaluated for VUR using VCUG and detected VUR was graded according to the International Reflux Study Committee from grade I to V (Figure 1) [5]. Based on the patient classifications used in previous studies, who considered grades I–III as low-grade VUR and grades IV and V as high-grade VUR [6]. Moreover, we reviewed patients' ultrasounds carried out at presentation, and hydronephrosis was graded according to the Society for Fetal Urology (SFU) system [7]. At our institution, a DMSA scan is performed in the case of FUTI, VUR grade IV–V, or suspected renal scar using ultrasound. A DMSA scan was performed 3 to 6 months after the last UTI, and those who had a DMSA renal scan before 6 months were excluded from the study. A DMSA scan was repeated in the case of recurrent FUTI. A gamma camera equipped with a low-energy, high-resolution collimator 2–3 hours after intravenous injection of a dose of 99 MTC DMSA [8]. The relative uptake function of both kidneys was calculated as the percentage renal uptake of each kidney. Image interpretation was assessed by the same team of radiologists in our hospital and was based on renal size, relative uptake function, uniformity of renal uptake, single or multiple cortical defects, and associated contraction or volume loss in the involved cortex.

**Figure 2:**
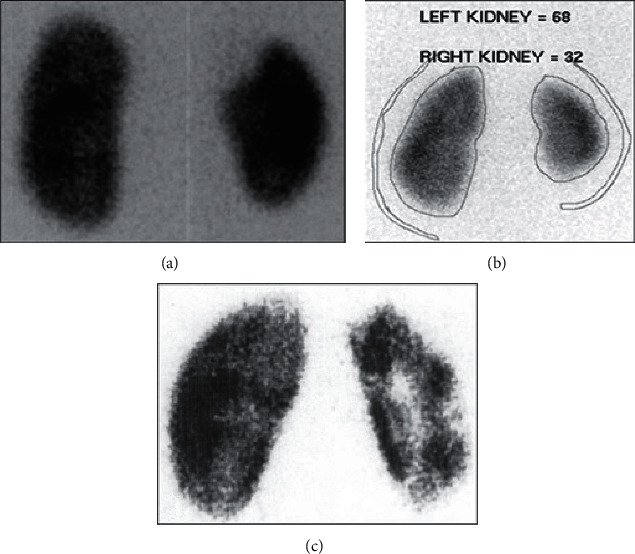
(a) DMSA scan image reveals a cold spot in the upper pole of the right kidney due to renal scarring in a child with a history of febrile urinary tract infections. (b) DMSA scan image reveals left multiple cold spots in the right kidney due to multiple renal scarring after recurrent urinary tract infections. (c) DMSA scan image reveals global atrophy of the right kidney due to grade V severe vesicoureteral reflux.

Reflux nephropathy was defined by Yu et al. [9] as any type of renal parenchymal abnormality associated with reflux, whether acquired due to UTIs or congenital dysplasia. We classified scarring according to Polito et al. [10] into mild (focal defects only), moderate (an overall decrease in renal nucleotide uptake that was 20 to 40% of relative uptake), and severe (shrunken kidney with less than 20% of the uptake) (Figure 2). Observational therapy with prophylactic antibiotics (trimethoprim plus or minus sulfamethoxazole or nitrofurantoin) was used initially in all patients since the time of the diagnosis of reflux. In general, patients were followed for 4 to 6 years. Indications for surgery were two FUTIs, breakthrough UTIs during observational therapy with prophylactic antibiotics or worsening of hydronephrosis during follow-up. Worsening hydronephrosis was defined as the upgrading of hydronephrosis. The type of surgery was chosen according to the renal function, laterality, and grade of VUR. Surgical options included extravesical ureteral reimplantation for high-grade reflux (grade IV–V) and endoscopic subureteral hyaluronic acid/dextranome (deflux) injection for low-grade reflux (grade I–III) as a double HIT technique [11]. Grade I is injected when there is a deterioration of reflux with an increasing grade of hydronephrosis. The criteria for selection for the type of technique either intravesical ureteral reimplantation or extravesical ureteral reimplantation were based on the degree of reflux, surgeon preference, and experience with the technique as it is a retrospective study. An extravesical procedure (Lich–Gregoir technique) is planned according to surgeon preference when the endoscopic procedure fails [12]. In high-grade VUR, infants are followed up to the age of two years. If VUR deterioration or renal scarring is seen, intervention is required. Our primary objective was to detect various factors that contribute to the incidence of renal scarring and their effects on the severity of scarring.

## 3. Statistics

The data were statistically analyzed using SPSS V26 (Statistical Package for Social Science). The categorical data were presented in numbers and percentages and evaluated using the Chi-square test. Medians and ranges were used to present continuous data, and nonparametric tests were used for evaluation. Univariate and multivariate analyses for possible independent predictors of renal scars were performed using COX regression. In the univariate analysis, only parameters with a *p*value of less than 0.1 were qualified for the multivariate analysis. A *p*value < 0.05 was considered statistically significant.

## 4. Results

**Figure 3:**
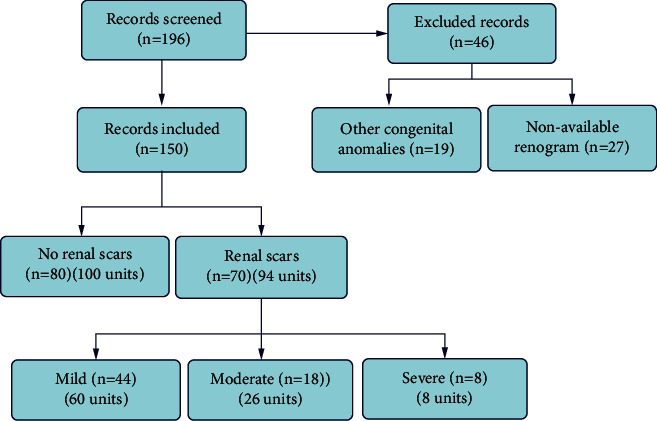
Flowchart of the study.

After reviewing the charts of 196 patients, we excluded 46 patients. Patients were excluded due to associated other congenital anomalies (19 patients) and nonavailable renogram studies (27 patients). Finally, we collected 150 patients (194 units) who were diagnosed with VUR at our institution. 80 patients (100 units) had no renal scars, while 70 patients (94 units) were diagnosed with renal scars (Figure 3). Patients' characteristics are presented in Table 1. It was noticed that the number of females was much higher than males; however, this was not statistically significant (*p*=0.053). The median follow-up duration was 54.2 months (14.2–109). The majority of patients in the [no renal scar group] presented with afebrile UTI, while those in the [renal scar group] presented mainly with FUTI (*p* < 0.001). Fifty percent of the [no renal scar group] had low-grade VUR (grades I and II), while most patients of the [renal scar group] had moderate or severe VUR (grades III–V) (*p* < 0.001). Patients of the [renal scar group] had more incidence of recurrent FUTI and a higher need for surgical intervention (*p* < 0.001for both). There was no significant difference between both groups regarding the age at surgery (*p*=0.19). Thirty-five units (35%) of the no scar group required surgical interventions (22 units had deflux injection and 13 had reimplantation). In the scar group, nearly all patients underwent surgical interventions (58 units had deflux injections, 32 had ureteral reimplantation, and 3 had nephrectomies). Laparoscopic simple nephrectomies were performed due to nonfunctioning kidneys with persistent pyuria. One patient in the scar group underwent conservative management because the scar was mild. Moreover, this patient did not experience recurrent FUTI throughout the follow-up. Regarding the degree of renal scarring, 60 units had mild scarring, 26 had moderate, and severe scarring was observed in 8 units (Table 2). In the [renal scar group], most kidneys with SFU grade 0 had mild scarring, 20/29 (69%) in comparison to 6 kidneys (75%) with severe scars that had high-grade hydronephrosis (SFU grades 3 and 4) (*p* < 0.001). Notably, all patients with severe renal scarring experienced recurrent FUTI in comparison to 83.3% with moderate scars and 52.3% with mild scars (*p* < 0.001). The univariate analysis, using COX regression, revealed that laterality, side, mode of presentation, the grade of VUR, and the incidence of recurrent FUTI were associated with the incidence of renal scarring. Using COX multivariate analysis, low-grade VUR (HR = 0.39, *p*=0.04), high-grade VUR (HR = 1,*p*=0.002), and FUTI recurrence (HR = 1.43, *p* = 0.03) were shown to be independent predictors of renal scarring (Table 3).

## 5. Discussion

Urinary tract infection (UTI) is the most common bacterial infection in pediatric patients. In the first 6 years of life, 8.4% of girls and 1.7% of boys will have a UTI [1]. UTIs cause fever, dysuria, and pain and may also result in permanent scarring of the kidney. Many factors, such as age, gender, race, and circumcision status, are risk factors for developing recurrent UTIs. [13]. Recurrent attacks of infection and vesicoureteral reflux (VUR) are major risk factors for the development of renal scarring [14]. Early diagnosis and treatment may prevent or decrease renal scarring caused by acute febrile UTI [15]. Reflux nephropathy lesions are often present in neonates with high-grade VUR in the absence of UTIs. It has been reported that 71% of uninfected neonates with high-grade VUR had renal scarring detected by renal isotope scanning [16]. The DMSA renal scan is considered by many authors as the most sensitive modality for detecting renal scarring. Studies reveal that 30 to 50% of children with VUR have renal parenchymal scarring [17]. The presented data did not confirm this belief. The authors did not find a correlation between age groups and sex with VCUG or DMSA renal scan results. In our study, in a selected group of patients who underwent antireflux surgery, it was noted that the grade of VUR is a predominant feature in the association of scars. The mode of presentation cannot detect the presence or absence of scars. The presence of UTIs may not denote the presence of associated scarring. However, treatment of high-grade VUR before the development of UTIs may protect against renal scarring. Early treatment in cases of tubular scarring correlates with reversal of the corresponding abnormalities observed on Tcm 99-DMSA scanning [17]. Moreover, the data from Orellana et al. [18] found that children older than 1 year developed more renal scarring after pyelonephritis than those younger than 1 year (70.1% vs. 36.4%;*p* <  0.0001), which is in contrast to most reports. Acute pyelonephritis without renal scarring has been reported [19]. Severe forms of VUR were associated with dysmorphic kidneys.

Merguerian et al. [20] reported a correlation between renal scarring and age. Renal abnormalities were present in 20% of infants younger than one year of age compared with a 5% incidence of focal scars. The incidence of focal scarring is higher after the age of one year (16% and 20%). In infants younger than one year of age, imaging of dysmorphic kidneys suggested a congenital basis for nephropathy [16]. However, focal scarring is strongly correlated to the VUR of infected urine in children older than one year. Fifty percent of reflux-associated nephropathy presents as focal scarring. In our study, 44 patients (60 units) have focal scarring, and 8 patients (8 units) had global atrophy and less than 20% function. Focal scars may indicate previously missed infections, and global atrophy may denote a congenital basis that correlates well with Merguerian et al. [20] results. In our study, a female predominance was noted in patients who had scars with a 1 : 3 male to female ratio. On the other hand, Ransley [21] reported that sterile VUR itself does not cause renal scarring in pigs. In clinical practice, the evidence is largely circumstantial, but conservative management of patients with VUR is based on the recognition that scarring does not occur when sterile urine is maintained with antibacterial prophylaxis [22]. Mackie and Stephens [23] proposed that renal dysplasia and/or hypoplasia are related to the abnormal development of the embryonic ureteral bud. Further terminological confusion is caused by reports of acute pyelonephritis followed by renal scarring in the absence of VUR [24]. In our study, approximately 50% of patients had scars at the first presentation of VUR, and new scars developed in the previously unscarred kidneys as reported by the International Reflux Study Committee irrespective of medical or surgical treatment of VUR [25]. These observations could be proposed for a prospective study for screening children susceptible to VUR. The development of reflux nephropathy and renal scarring is a multifactorial process. Risk factors for renal scarring include VUR, UTI, and previous scar/dysplasia [26]. Although antenatal US can help identify up to 30% of newborns with VUR, exposing detectable renal damage before UTI, US imaging can miss approximately 50% of the scars. In our study, 46.7% of our patients had abnormal DMSA. Our study revealed that renal scars are more associated with the grade of VUR than UTIs. Researchers from Sweden reported a higher prevalence of scars in more severe grades [27]. The prenatal ultrasound can play an important role in the early detection of VUR. Further postnatal evaluation reveals VUR in 10 to 30% of patients with hydronephrosis on ultrasound [28]. It is reported that the incidence of VUR in siblings of patients is higher than in the general population. VUR in asymptomatic siblings is of low grade, in contrast to symptomatic VUR, which is of high grade and associated with a higher incidence of reflux nephropathy [29]. The aim of early detection of VUR or any intervention is to prevent upper tract damage. Our findings encourage the early intervention of high-grade VUR to prevent renal damage.

The present study results are consistent with many other studies that confirmed the presence of a correlation between developing renal scarring and the degree of severity of the coexisting VUR. Camacho et al. [30] reported that children with normal DMSA had a lower chance of VUR than children with abnormal DMSA (12% vs. 48%). Hoberman et al. [13] reported that renal scarring was less likely to occur in children without VUR compared with those with VUR (6% vs. 14.7%,*p*=0.03). A large portion of patients with renal scarring in the absence of demonstrable reflux suggests that other mechanisms, such as bacterial adherence, may play a role in bacterial transportation to the kidney. Supavekin et al. [31] concluded that younger children have a risk of permanent renal damage similar to that noted in older children.

In our study, we confirmed the correlation between recurrent UTI and VUR with renal scarring on DMSA renal scintigraphy. We recommend that all children with recurrent UTI and/or VUR, irrespective of age and sex, will benefit from DMSA renal scintigraphy to detect permanent renal scarring. Prospective studies are needed to provide more complete data to demonstrate renal scarring with prognosis and outcome. Antireflux surgery does not prevent progressive renal scar development and renal functional deterioration. This is true, especially in children with bilateral renal scarring. If there is indeed a lack of preventive benefit with continuous antibiotic prophylaxis and/or antireflux surgery for acute pyelonephritis and progressive renal injury in children with VUR, one may question the value of treating or even diagnosing VUR. We did not study the effect of antireflux surgery on the prevention of renal scarring, but it will need further evaluation [32]. Medical treatment and surgical correction of VUR do not effectively prevent recurrent UTI and new scar formation and progression to end-stage renal disease. Thus, physicians should focus on the prevention of initial renal scarring [33].

Limitations of the present study include possible sampling bias due to the retrospective nature of the study. Also, we did not study the effect of antireflux surgery on the prevention of further renal scarring.

## 6. Conclusion

Approximately 50% of patients who underwent antireflux surgery had renal scarring at presentation. Renal scarring is more linked to the grade of VUR and UTIs. We advocate proper investigation and management of infants who have had UTIs with or without fever for early detection of VUR, which may play a role in the prevention of acquired renal scarring.

## Figures and Tables

**Table 1:** Patients' characteristics.

Parameter	No renal scar group, 80 patients 100 units	Renal scar group, 70 patients 94 units	*p*-value
Age at presentation, median (range)	11.3 months (0.1–28.4)	10.8 months (0.1–21.3)	0.32
Gender Male, n (%) Female, n (%)	30 (37.5) 50 (62.5)	16 (22.9) 54 (77.1)	0.053
Laterality Unilateral, n (%) Bilateral, n (%)	60 (75) 20 (25)	46 (65.7) 24 (34.3)	0.21
Side Right, n (%) Left, n (%)	26 (26) 74 (74)	28 (29.8) 66 (70.2)	0.56
Mode of presentation AHN, n (%) Febrile UTI, n (%) Afebrile UTI, n (%)	10 (12.5) 20 (25) 50 (62.5)	10 (14.3) 46 (65.7) 14 (20)	<0.001
SFU grade SFU grade 0, n (%) SFU grade 1 + 2, n (%) SFU grade 3 + 4, n (%)	33 (33) 65 (65) 12 (12)	29 (30.9) 47 (50) 18 (19.1)	0.21
VUR grade Grade 1 and 2, n (%) Grade 3, n (%) Grades 4 and 5, n (%)	50 (50) 28 (28) 22 (22)	10 (10.6) 40 (42.6) 44 (46.8)	<0.001
Recurrent FUTI, n (%)	12 (15)	52 (74.3)	<0.001
Need for surgery, n (%)	35/100 (35)	93/94 (98.9)	<0.001
Age at surgery, mean□SD	38.6 months (17.4 – 59.9)	34.1 months (11.6 – 65.8)	0.19

FUTI: febrile urinary tract infections, AHN: antenatal hydronephrosis, SFU: Society of Fetal Urology, and VUR: vesicoureteral reflux.

**Table 2:** Distribution of patients with degree of renal scarring

	Renal scarring, 94 renal units			*p*-value
	Mild, 60 units	Moderate, 26 units	Severe, 8 units	
Age at presentation, median (range)	10.3 mon (0.1–19.2)	11.2 mon (0.2–21.3)	9.1 mon (0.1–20.9)	0.25
Gender Male, n (%) Female, n (%)	10 (22.7) 34 (77.3)	4 (22.2) 14 (77.8)	2 (25) 6 (75)	0.99
Laterality Unilateral, n (%) Bilateral, n (%)	28 (63.3) 16 (36.4)	10 (55.6) 8 (44.4)	8 (100) 0 (0)	0.08
Side Right, n (%) Left, n (%)	16 (26.7) 44 (73.3)	11 (42.3) 15 (57.7)	1 (12.5) 7 (87.5)	0.19
Mode of presentation FUTI, n (%) Afebrile UTI, n (%) AHN, n (%)	30 (68.2) 7 (15.9 (15.9)	12 (66.7) 4 (22.2) 2 (11.1)	4 (50) 3 (37.5) 1 (12.5)	0.7
SFU grade SFU 0, n (%) SFU 1 and 2, n (%) SFU 3 and 4, n (%)	20 (33.3) 33 (55) 7 (11.7)	8 (30.8) 13 (50) 5 (19.3)	1 (12.5) 1 (12.5) 6 (75)	0.001
Grade of VUR Grade I–II, n (%) Grade III, n (%) Grade IV–V, n (%)	10 (16.7) 30 (50) 20 (33.3)	0 (0) 10 (38.5) 16 (61.5)	0(0) 0(0) 8 (100)	0.005
Recurrent FUTI	23 (52.3)	15 (83.3)	8 (100)	0.006

FUTI: febrile urinary tract infections, AHN: antenatal hydronephrosis, SFU: Society of Fetal Urology, and VUR: vesicoureteral reflux

**Table 3:** Univariate and multivariate analysis to evaluate the possible independent predictors of renal scarring

Parameter	Univariate analysis			Multivariate analysis		
	HR	CI	*P* value	HR	CI	*P* value
Age at presentation	0.67	0.29–1.09	0.22	—	—	—
Gender	0.89	0.65–1.22	0.46	—	—	—
Laterality	0.81	0.6–1.02	0.07	1.313	0.97–1.78	0.12
Side	1.67	0.94–2.4	0.09	1.61	0.74–3.53	0.22
Presentation						
FUTI	1	—	0.03	1	—	0.09
Afebrile UTI	0.73	0.49–0.97	0.04	0.72	0.23–2.25	0.17
AHN	0.18	0.05–0.31	0.012	0.26	0.002–2.99	0.15
SFU grade						
Grade 0	0.75	0.01–1.49	0.6	—	—	—
Grade 1 and 2	1.2	0.94–1.45	0.56			
Grade 3 and 4	1	—	0.42			
VUR grade						
Grade 1and 2	0.27	0.12–0.43	0.01	0.39	0.19–0.95	0.04
Grade 3	0.69	0.43–95	0.03	0.66	0.29–1.03	0.14
Grade 4 and 5	1	—	0.005	1	—	0.02
Recurrent UTI	2.65	2.15–3.15	0.002	1.43	1.03–1.83	0.03

FUTI: febrile urinary tract infections, AHN: antenatal hydronephrosis, SFU: society of fetal urology.

## Data Availability

The datasets used and/or analyzed during the current study are available from the corresponding author on reasonable request.

## Abbreviations

VUR: Vesicoureteral reflux
DMSA: Dimercaptosuccinic acid
VCUG: Voiding cystourethrography
UTIs: Urinary tract infections
FUTIs: Febrile urinary tract infections
AHN: Antenatal hydronephrosis
SFU: Society for Fetal Urology
Double HIT Technique: Double hydrodistention implantation technique.
